# MOTIPS: Automated Motif Analysis for Predicting Targets of Modular Protein Domains

**DOI:** 10.1186/1471-2105-11-243

**Published:** 2010-05-11

**Authors:** Hugo YK Lam, Philip M Kim, Janine Mok, Raffi Tonikian, Sachdev S Sidhu, Benjamin E Turk, Michael Snyder, Mark B Gerstein

**Affiliations:** 1 Program in Computational Biology and Bioinformatics, Yale University, New Haven, CT 06520, USA; 2 Department of Molecular Biophysics and Biochemistry, Yale University, New Haven, CT 06520, USA; 3 Department of Molecular, Cellular and Developmental Biology, Yale University, New Haven, CT 06520, USA; 4 Department of Molecular Genetics, University of Toronto, Toronto, Ontario, M5S 1A8, Canada; 5 Banting and Best Department of Medical Research, University of Toronto, Toronto, Ontario, M5G 1L6, Canada; 6 Department of Pharmacology, Yale University, New Haven, CT 06520, USA; 7 Department of Computer Science, Yale University, New Haven, CT 06520, USA; 8 Current Address: Terrence Donnelly Centre for Cellular and Biomolecular Research, University of Toronto, Toronto, Ontario, M5S 3E1, Canada; 9 Current Address: Stanford Genome Technology Center, Department of Biochemistry, Stanford University, Palo Alto, CA 94304, USA; 10 Current Address: Department of Genetics, Stanford University, Palo Alto, CA 94305, USA

## Abstract

**Background:**

Many protein interactions, especially those involved in signaling, involve short linear motifs consisting of 5-10 amino acid residues that interact with modular protein domains such as the SH3 binding domains and the kinase catalytic domains. One straightforward way of identifying these interactions is by scanning for matches to the motif against all the sequences in a target proteome. However, predicting domain targets by motif sequence alone without considering other genomic and structural information has been shown to be lacking in accuracy.

**Results:**

We developed an efficient search algorithm to scan the target proteome for potential domain targets and to increase the accuracy of each hit by integrating a variety of pre-computed features, such as conservation, surface propensity, and disorder. The integration is performed using naïve Bayes and a training set of validated experiments.

**Conclusions:**

By integrating a variety of biologically relevant features to predict domain targets, we demonstrated a notably improved prediction of modular protein domain targets. Combined with emerging high-resolution data of domain specificities, we believe that our approach can assist in the reconstruction of many signaling pathways.

## Background

Important protein-protein interactions (e.g., those involved in signal transduction) are often mediated by modular protein domains [1]. These domains often work in a mix-and-match fashion, thereby acting as the building blocks of signaling pathways [2]. Examples include the SH3 and WW domains that bind proline-rich motifs [3], and the serine/threonine kinase domain that specifically phosphorylates the hydroxyl group of serine and threonine [4]. Throughout we will refer to these collectively as "domains". Since these kinds of domains play an important role in the assembly, regulatory and signaling activities of the cell [3,5,6], accurate prediction of their targets is crucial to understanding many biological pathways [7,8].

As a result, various techniques have been developed to predict domain targets and to enhance the prediction. Earlier studies have tried to use consensus sequences from phage display experiments to predict the targets of peptide-binding domains [9]. Also, a modern peptide library screening approach, which is commonly used to determine phosphorylation motifs for kinases, has shown to have high accuracy in determining domain specificity [10]. Both approaches have in common that they identify the specificity of each domain in a position-specific manner, yielding a Position Specific Scoring Matrix (PSSM; also known as Position Weight Matrix, PWM). Furthermore, many studies have demonstrated various ways to improve prediction performance using genomic information. For instance, comparative genomics and secondary structure information have been used to increase the performance of SH3 target prediction [11,12].

Nevertheless, to date the prediction of biologically relevant targets of these domains has yet to be addressed in an automated and integrated fashion. To this end, we present an automated process, which integrates comparative genomic (i.e., sequence conservation) and structural genomic (i.e., surface propensity and peptide disorder) data with traditional profile scanning method to predict domain targets based on experimental screening result (e.g. peptide library screening) or their derived PSSMs. The process is fully automated and implemented as an online server. The implementation is open-source and also available for download at http://motips.gersteinlab.org.

## Results and Discussion

### An Automated Pipeline Process

Our approach first converts the input data into a PSSM and then normalizes it. Secondly, it scans the target proteome by using the normalized PSSM and generates a hit list of potential domain targets. Following the motif scanning, it computes the conservation score, solvent accessibility score, and disorder score for each motif hit based on the pre-computed scores for each protein residue. It then integrates these genomic features with the motif matching scores and the number of hits per protein by naïve Bayes to predict the optimal targets based upon a validated training set. Lastly, it sorts the motif hits by their likelihood of having interaction with the domain and consolidates them into unique protein hits.

### Data Conversion and Normalization

**Figure 1:**
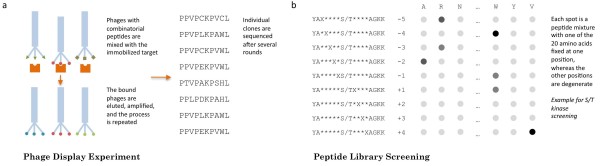
**Experiments for Motif Identification**. a) The phage display experiment identifies potential target peptides of short sequences, and b) the peptide library screening measures the binding specificity at position level. The resulting experimental data of such experiments can be converted into a Position Specific Scoring Matrix (PSSM).

A number of experimental approaches, such as phage display and peptide library screening (see Figure 1), have been developed to identify domain binding and phosphorylation targets. However, data from different experiments result in different formats that always complicate the data analysis process. To keep the process consistent and standardized, these data are converted into PSSM followed by normalization (for supported input formats, see System Implementation and Availability).

Our approach employs two different ways to normalize the input data. The first approach is designed for signal data from experiments such as from peptide library screening. It normalizes the signal score for each amino acid at each position by the following equation:(1)

where *Z*_*ca *_is the normalized score for amino acid *a *at position *c*, which has a signal score *S*_*ca*_, and *m *is the total number of amino acids. Equation (1) thus computes the weight for each amino acid at each position and scales it up by the total number of amino acids. However, to consider the known specificity for domains such as the serine/threonine kinase domain, which have fixed amino acid targets (e.g., serine and threonine) at a certain position in the binding motif, a score of 0 is automatically assigned to every other amino acid that is not expected at that position. To indicate the slight probability of observing the fixed amino acids at other positions, a pseudo-count of 1 is assigned to each of them at these non-specific positions.

The second way of normalization is designed for peptide data from experiments such as from phage display experiment. Our approach employs the pseudo-count method based on substitution probabilities to complement the incomplete or imperfect representation of a position in the original peptide data [13]. Pseudo-counts are needed since this kind of experiments significantly undersample sequence space, thereby severely penalizing rare residues. It calculates the probability *p*_*ca *_of amino acid *a *at position *c *by equation (2) as follows:(2)(3)

where *n*_*ca *_and *b*_*ca *_are the count and pseudo-count for amino acid *a *at position *c*, while *N*_*c *_and *B*_*c *_are the total count and pseudo-count for all amino acids. The total pseudo-count *B*_*c *_is calculated from equation (3) with *ψ *as an empirically chosen positive number (default to 5) and *R*_*c *_as the unique count for all amino acids at position *c*. Taking different substitution probabilities of different amino acids into consideration, substitution matrixes such as the BLOSUM 62 [14,15] and McLachlan [16] matrixes are used to calculate pseudo-count *b*_*ca *_by equation (4) shown as the following:(4)

where *q*_*ia *_is the substitution probability for amino acid *a *replaced by *i*, and *Q*_*i *_is the substitution probability for *a *replaced by any amino acid. In addition to the pseudo-count method based on substitution probabilities, we also provide alternative pseudo-count methods based on flat counting (adding 1 to all values) and entropy (adding a pseudo-count proportional to the entropy of each position to its corresponding values).

### Motif Scanning and Scoring

**Figure 2:**
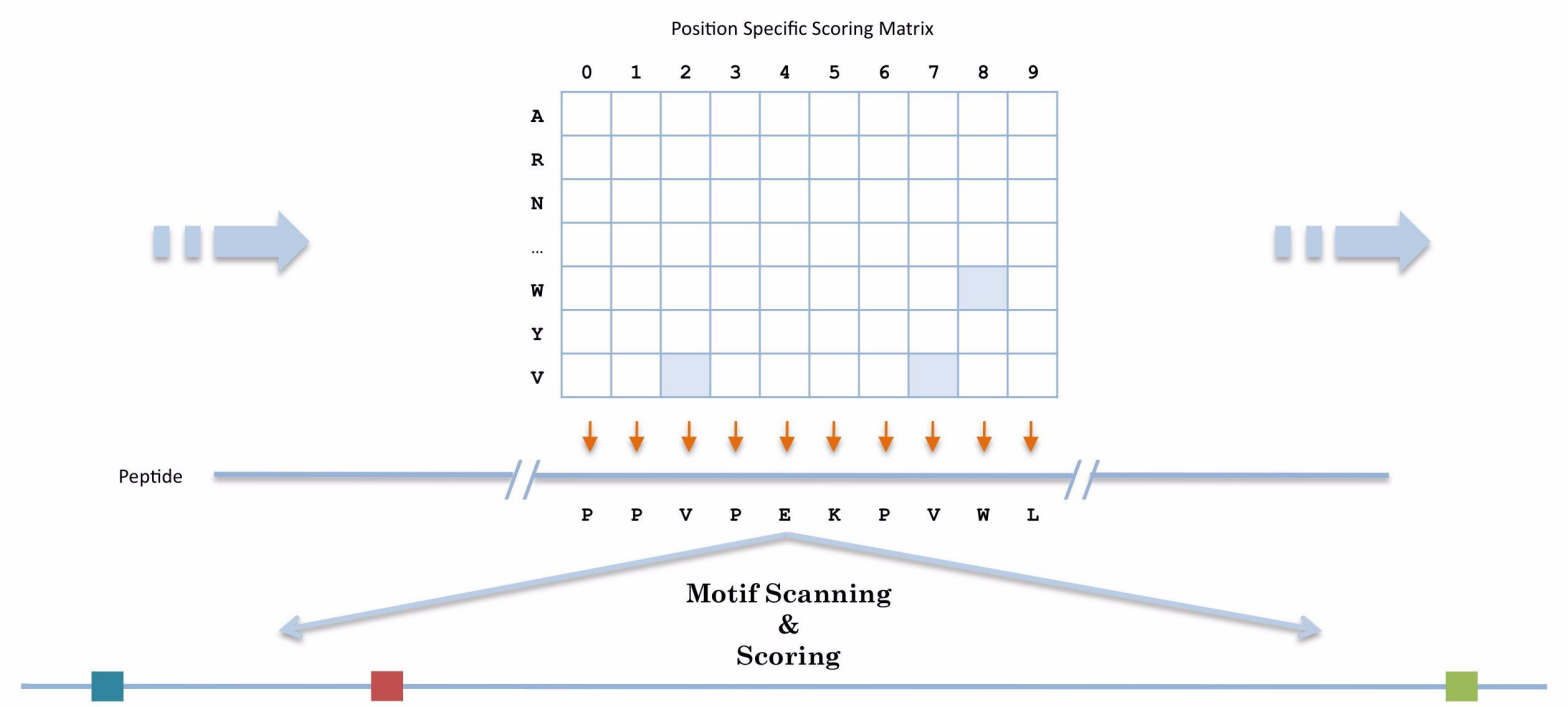
**Motif Scanning and Scoring**. Identify potential target sites of the domain by sliding a Position Specific Scoring Matrix (PSSM) across the peptides in the proteome and comparing the motif matching scores for each window.

To scan the target proteome for potential domain targets and to score them, our approach uses a window-sliding method based on a normalized PSSM similar to the method used in Scansite [17,18]. For each protein in the target proteome, it slides a window of size equivalent to the length of the motif on the peptide sequence by every single amino acid (see Figure 2). Based on the scoring matrix, the score for each window sequence is calculated by equation (5):(5)

where *l *is the length of the motif and *S*_*ca *_is the score for amino acid *a *at position *c *in the window sequence. This equation is also used to calculate an optimal score of the motif where *S*_*ca *_is the maximum score at position *c *in the scoring matrix. Then the final normalized score *E *for the window sequence is calculated by equation (6):(6)

To improve the efficiency of the scanning algorithm, each motif hit is compared immediately to a sorted hit list of fixed size (currently 2,000 hits) and will only be retained if it has a more significant score than the least significant one in the list.

### Structural Features and Scoring

**Figure 3:**
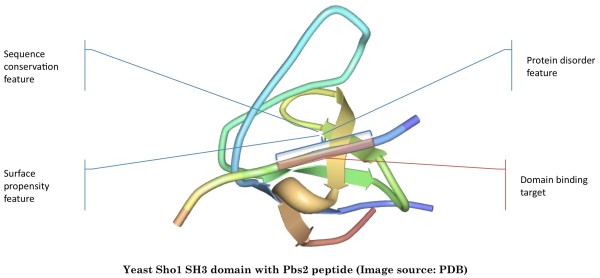
**A Peptide-Binding Domain Example**. A peptide-binding domain, such as the SH3 domain, recognizes the binding site on a peptide which exhibits certain structural and conservation features including surface propensity, protein disorder, and sequence conservation.

Although a profile-matching scan could identify possible domain targets, it does not take into account the structural information of the target sequences that are also related to protein-protein interactions. For instances, sequences exposed on the surface should be more accessible than those that are buried; sequences that are unfolded should be more easily bound than those that are folded; and structures that are highly conserved among close species could have more biological significance. Taking these factors into account, our approach includes three major structural and conservation features in the prediction, which are surface propensity, protein disorder, and sequence conservation, to complement the motif scanning score (see Figure 3).

The degree of surface propensity of a given sequence is measured by its relative solvent accessibility, which represents the extent of residue solvent exposure. It is predicted by a protein structure prediction program, SABLE, which uses a neural network-based regression algorithm [19]. To measure the disorder of the sequence, DISOPRED, a neural networks and PSI-BLAST-based approach is used to estimate the probability of the region being disordered [20,21]. For measuring the conservation of the sequence structure, orthologs of the sequence are identified using INPARANOID [22]. Following the ortholog identification, the sequences in the orthologous groups are aligned with MUSCLE [23] and a conservation score for each position in the sequence is estimated by its entropy using AL2CO [24].

For each protein in each proteome being studied, the solvent accessibility, disorder and conservation scores are pre-computed for each residue. As a result, the scores for the motif hits could be calculated in a timely manner.

### Feature Integration and Target Prediction

In addition to calculating the structural and conservation scores for each motif hit, the number of hits per protein is also calculated as a feature for the hit. Our approach then applies a Bayesian learning algorithm to integrate all the aforementioned features, including the motif scanning score, solvent accessibility score, disorder score, conservation score, and number of hits per protein, to predict potential domain targets. Because of the simplicity and efficiency of the naïve Bayes model, it is employed to build a classifier based on a validated training set under the assumption of independence of the features. In particular, the default models (i.e., the SH3 model based on Sho1 and the S/T kinase model based on Prk1) used a number of experimentally determined interaction pairs [25,26] as the gold-standard positives to train the algorithm. Moreover, a set of paired proteins in which each pair was annotated to always localize to two different compartments (for example, nucleus only and cytoplasm only in the Gene Ontology) in the cell was selected as the gold-standard negatives. The conditional probability can then be calculated from the given features based on equation (7):(7)

where *I *is the class variable (i.e., interaction or non-interaction), *F *is the feature such as the motif scanning score, and *n *is the total number of features. To assess the independence of the features, pair-wise correlation coefficients were calculated. The results showed the pair-wise correlation coefficients have an average of 0.23 for the SH3 model and 0.18 for the S/T kinase model, indicating the features are to a large extent independent. Furthermore, since the independency assumption is not harmful for data pre-processed with Principal Component Analysis (PCA) [27], we performed PCA to transform the possibly correlated features into uncorrelated features. The first three principal components were chosen to build a naïve Bayes model followed by a stratified 10-fold cross-validation. The Area Under Curve (AUC; 89.1 for the SH3 model and 75.9% for the S/T kinase model) of the Receiver Operating Curve (ROC) resulting from the PCA transformation was then compared to the AUC (91.8% for the SH3 model and 78.6% for the S/T kinase model) without PCA. No significant deviation of performance was observed between the predictions without PCA and those with PCA, indicating no strong dependency among the original features.

Finally, the motif hits from the domain of interest are classified under the selected model and sorted by their likelihood of having an interaction with the domain. Hits for the same protein are consolidated into one single hit represented by the most likely target. Genomic information that is not used in the prediction, such as protein-protein interaction data, localization data and phosphorylome data, could also be integrated easily with the tab-delimited hit list for further analysis while phosphorylation prediction data from mass spectrometry experiments can be used as cross-validation.

### Prediction Performance

**Figure 4:**
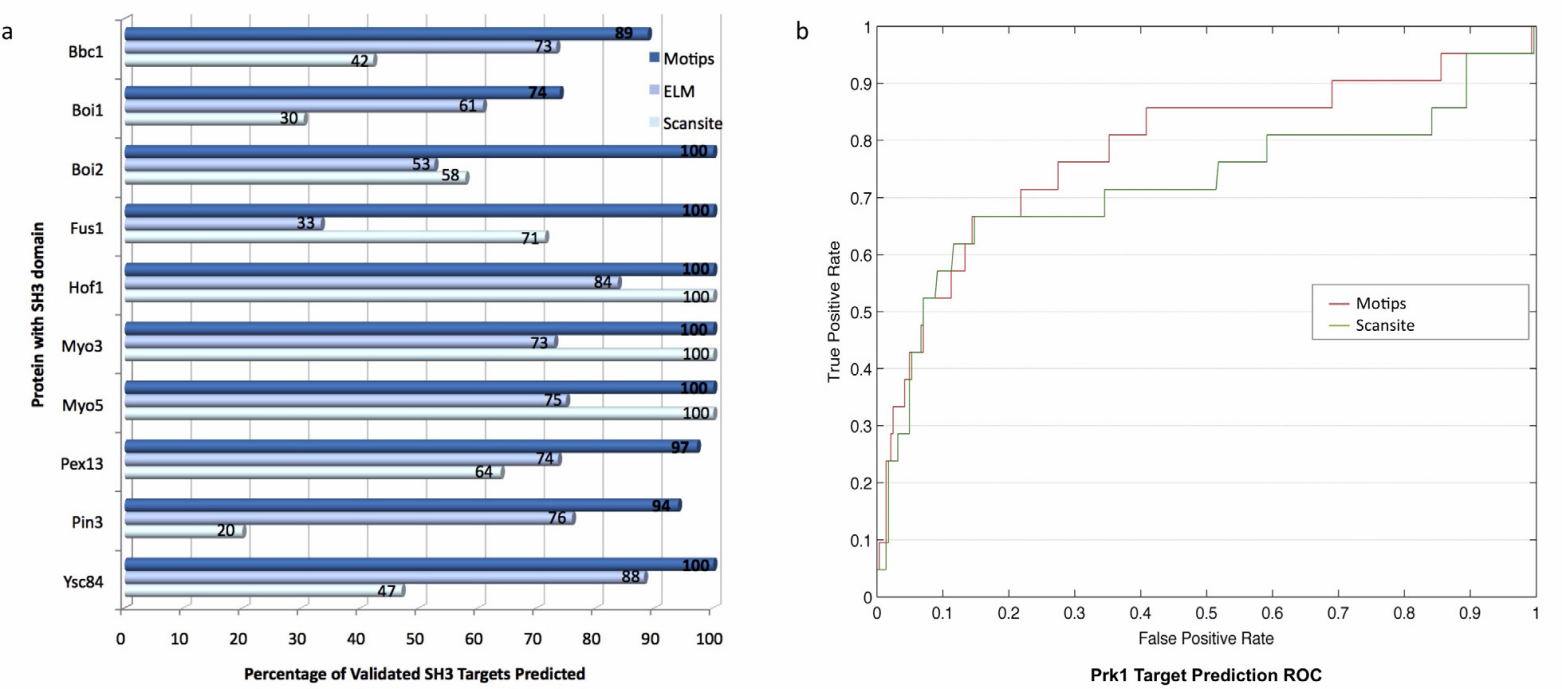
**Targets Prediction Performance**. a) The benchmark of SH3 target prediction based on the validated targets for 10 different SH3 proteins, and b) the Receiver Operating Curve (ROC) comparing the prediction performance for the Prk1 kinase targets.

To assess the prediction performance of our approach, we benchmarked with two existing methods: 1. the Eukaryotic Linear Motif (ELM) database [28], which predicts functional sites in eukaryotic proteins by patterns with context-based rules and logical filters such as the structure filter; and 2. the Scansite method [17], which uses a motif profile-scoring approach to predict sites within proteins that are likely to be phosphorylated or bind to domains. Based on the SH3 interactome data [25], a model for the SH3 domain was trained with the Sho1 interactions. Then, we performed our prediction, requiring a likelihood value above 0.9, on 10 other different SH3 proteins by using the aforementioned model. We compared our results with the predictions from the ELM database (data retrieved from the web server using a Python program for 5 different SH3 ligands available on the server) and from the Scansite scanning (which requires a score not more than 3 fold of the optimal score). Our results (see Figure 4) show that on average our prediction has a 49% increase in accuracy in predicting the validated targets of the SH3 proteins when compared to the ELM prediction. When compared to the profile-scoring method of Scansite, our prediction is almost twice as accurate (90% higher). In addition to predicting SH3 targets, our approach was employed to predict Prk1 phosphorylation sites [26]. A stratified 10-fold cross-validation has shown a performance increase (see Figure 4; 79% AUC in a ROC curve) when compared to the profile-scoring method (72% AUC).

### System Implementation and Availability

The motif analyzing process mentioned above is implemented as an online server, which allows researchers to upload their experimental data representing the motifs of the domains and to predict the targets. Our pipeline supports various input data formats. For specific analysis software, it currently supports the Gene Pix Result format http://www.moleculardevices.com/pages/software/gn_genepix_file_formats.html#gpr that is usually used for peptide library screening data, and the BRAIN project's peptide format http://www.baderlab.com/Software/BRAIN/PeptideFile that is usually used for phage display experiments. For general purposes, it supports the FASTA format (i.e., a set of peptides with the same length that represent the possible interacting sites) and the Nx20 format (i.e., a tab-delimited format that represents the positional scores of a motif profile with the first row labeled with the amino acid residues and the subsequent rows as the different positions). The pipeline currently has a compilation of 20 proteomes consisting of 14 yeast proteomes (*S. cerevisiae*, *C. albicans*, *D. hansenii*, *C. glabrata*, *K. lactis*, *N. crassa*, *S. bayanus*, *S. castelli*, *S. kluyveri*, *S. kudriavzevii*, *S. mikatae*, *S. paradoxus*, *S. pombe*, *Y. lipolytica*), 2 worm proteomes (*C. briggsae*, *C. elegans*), and 4 mammalian proteomes (*C. familiaris*, *P. troglodytes*, *M. musculus*, *H. sapiens*).

The feature scores were pre-computed and the default prediction models, which could be replaced by a user-defined training set (a tab-delimited file with the gene on the first column and a logical value on the second indicating the interaction), were also built. Moreover, the analyzing process is implemented as an asynchronous multi-threading pipeline process so the prediction results can be delivered to the users via email offline, in addition to being displayed online. Furthermore, the entire system is built using the Java programming language under a Model View Controller architecture in which the analysis process is implemented as a standalone open-sourced program. Therefore, the process could be customized by researchers and executed in command line on multiple platforms. The naïve Bayes classification is performed using Weka, the open-source Java data mining software [29].

The standalone pipeline and database are available for download at the MOTIPS server at http://motips.gersteinlab.org.

## Conclusions

By integrating a variety of biologically relevant features and using a Bayesian learning algorithm to predict domain targets, our approach has improved the domain binding and phosphorylation target predictions notably compared to using only profile-matching scan. We believe our approach is versatile enough to predict targets of domains of different kinds, and its implementation as an online public server could facilitate researchers in predicting domain targets more accurately.

## Authors' contributions

HL and PK designed the methodology and drafted the manuscript. HL implemented the methodology. JM carried out the kinase specificity experiments and participated in its analysis. RT, SS, BT, MS and MG guided the study and helped to draft the manuscript. PK and MG conceived of the study. All authors read and approved the final manuscript.
